# Supplementary material for: A high-resolution mRNA expression time course of embryonic development in zebrafish
Source: eLife. 2017 Nov 16;6:e30860. doi: 10.7554/eLife.30860 (PMC5690287; doi:10.7554/eLife.30860)
Supplement: Supplementary file 6. [file elife-30860-supp6.zip › biolayout-clusters-files/Cluster240-genes.html]

Cluster240


# Cluster240: Genes

| Ensembl ID | Gene Name | Chr | Start | End | Biotype |
| --- | --- | --- | --- | --- | --- |
| ENSDARG00000091902 | b3gnt2b | 17 | 24018390 | 24063963 | protein\_coding |
| ENSDARG00000030972 | dnaja1 | 1 | 44301668 | 44313131 | protein\_coding |
| ENSDARG00000070675 | fam3a | 23 | 25209317 | 25223747 | protein\_coding |
| ENSDARG00000027589 | fzd7b | 6 | 9468182 | 9472060 | protein\_coding |
| ENSDARG00000102458 | hip1rb | 5 | 65687647 | 65750230 | protein\_coding |
| ENSDARG00000060169 | mns1 | 7 | 32849017 | 32859402 | protein\_coding |
